# Multiple-reaction monitoring (MRM) LC–MS/MS quantitation of venlafaxine and its *O*-desmethyl metabolite for a preclinical pharmacokinetic study in rabbits

**DOI:** 10.1038/s41598-022-13389-6

**Published:** 2022-06-04

**Authors:** Abdul Aala Fazli, Bala Krishna Panigrahy, Varinder Kumar, Syed Naiem Raza, Bilal Ahmad Zarger, Taha Umair Wani, Shavej Ahmad, Arshad Khuroo, Nisar Ahmad Khan

**Affiliations:** 1 Department of Pharmaceutical Sciences, University of Kashmir, Hazratbal, Srinagar, 190006 India; 2 Sun Pharmaceuticals Industries Limited (R&D Center), Gurugram, Haryana 122021 India; 3 Viatris Inc (Formulations Development Research), Jigani, Bangalore, 560105 India; 4 University Institute of Pharma Sciences, Chandigarh University, Mohali, Punjab 140413 India

**Keywords:** Drug delivery, Pharmaceutics, Pharmacology, Preclinical research

## Abstract

Preclinical pharmacokinetic (PK) studies in animal models during the formulation development phase give preliminary evidence and near clear picture of the PK behavior of drug and/or its dosage forms before clinical studies on humans and help in the tailoring of the dosage form according to the expected and requisite clinical behavior. The present work reports a first of its kind preclinical PK study on extended-release (ER) solid oral dosage forms of venlafaxine (VEN) in New Zealand White rabbits. The VEN is a highly prescribed and one of the safest and most effective therapeutic agents used in the treatment of different types of depression disorders worldwide. The multiple-reaction monitoring (MRM) LC–MS/MS method developed for this purpose demonstrated enough reliability in simultaneously quantitating VEN and its equipotent metabolite *O*-desmethylvenlafaxine (ODV) in rabbit plasma. The method described uses solid-phase extraction for sample preparation followed by an ultrafast LC–MS/MS analysis. The chromatographic separation was achieved isocratically with a predominantly polar mobile phase by employing RPLC. The triple quadrupole LC/MS/MS system operated in MRM mode used an ESI probe as an ion source in positive polarity. The validation results are within the permissible limits of US FDA recommendations and acceptance criteria for bioanalytical method validation.

## Introduction

The preclinical testing for drug release in extended-release (ER) oral solid dosage forms, which also include tablets and capsules, comprises in vitro dissolution and in vivo pharmacokinetic (PK) studies in suitable animal models. The animal model preferred should have the capacity to house the particular type of ER formulation under preclinical investigation^1–3^. The data obtained from preclinical PK studies gives preliminary evidence about drug absorption rates and sites, and, a possible mechanism of drug distribution, metabolism, and elimination. This preclinical PK data collected in animal models also help in screening prototype ER formulations to support the development and selection of an optimal dosage form for clinical PK studies in humans^4,5^.

**Figure 1:**
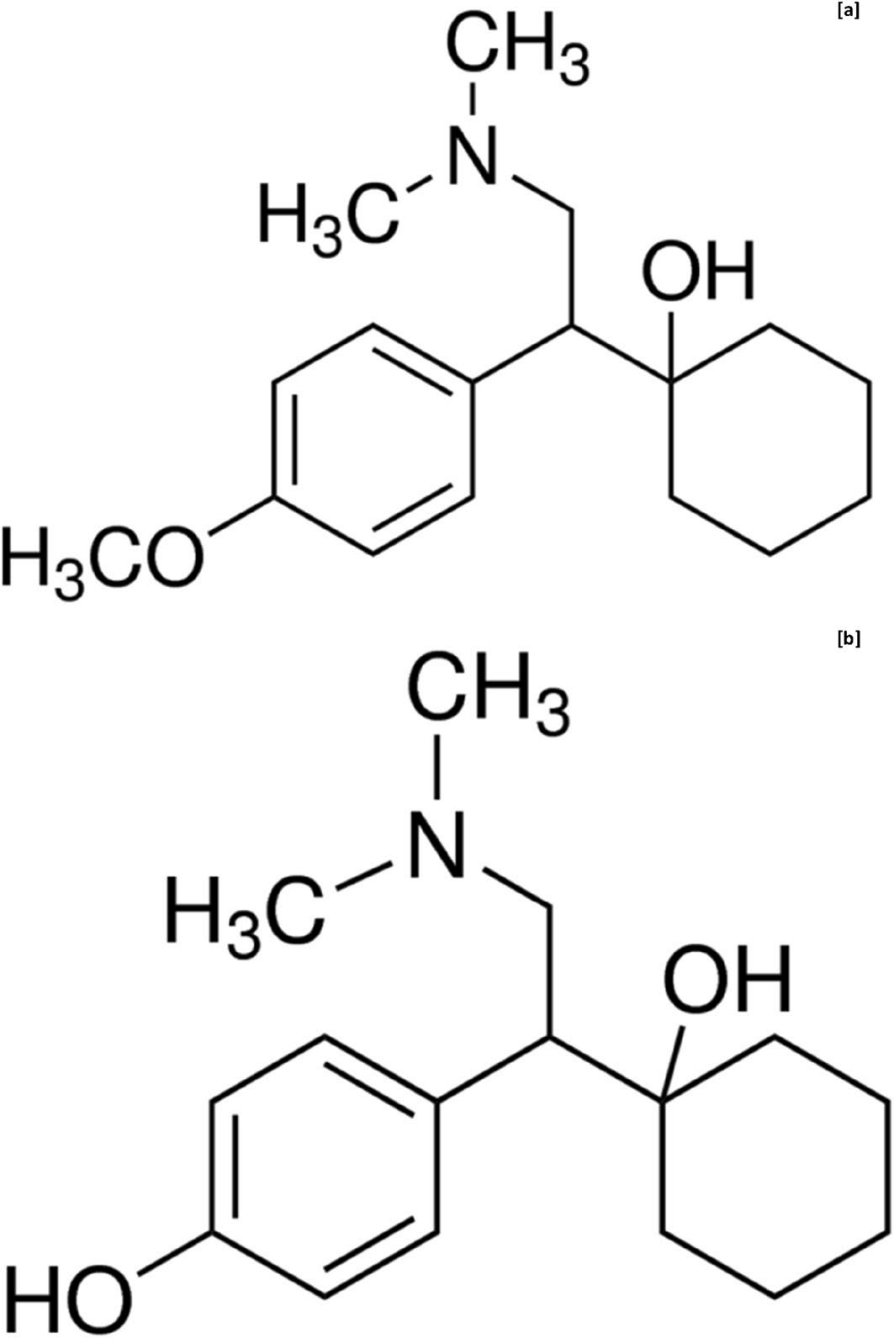
Chemical structure depiction of (**a**) venlafaxine and (**b**) *O*-desmethylvenlafaxine.

Venlafaxine (VEN), (*RS*) 1-[2-(dimethylamino)-1-(4-methoxyphenyl)ethyl]cyclohexanol, which belongs to the pharmacological class of serotonin noradrenaline reuptake inhibitors (SNRIs) is a relatively new and structurally novel antidepressant drug^6^ was introduced by Wyeth in 1993. It is chemically unrelated to tricyclic, tetracyclic, and other antidepressants^7,8^, and, is currently a highly prescribed and one of the most effective drugs with fewer unwanted side effects used in the treatment of depression disorders^9^. The VEN is administered orally in both immediate-release (IR) and ER dosage forms^10,11^. The antidepressant action of VEN and its major as well as an equipotent active metabolite, *O*-desmethylvenlafaxine (ODV)^12^ (Fig. 1) in humans is linked to their potentiation of neurotransmitter activity in the central nervous system. In human plasma, ODV predominates VEN^13^ in most people except for slow metabolizers, where VEN has been found in higher concentrations than ODV^14,15^. Both VEN and ODV are potent inhibitors of serotonin and noradrenaline reuptake, and also weakly inhibit the dopamine reuptake^16^. Venlafaxine being a racemate, the *R*-(−) and *S*-(+) enantiomeric forms are present in equal amounts and both contribute towards its antidepressant activity. The *R*-enantiomer of VEN is potent in inhibiting the synaptosomal serotonin and noradrenaline reuptake, while the *S*-enantiomer is more selective in inhibiting the serotonin reuptake. However, both the enantiomers of VEN are more potent in inhibiting the serotonin reuptake in contrast to that of noradrenaline^17–19^. The enantiomers of ODV also inhibit both noradrenaline and serotonin reuptake with *R*-enantiomer being more potent^20^.

Drug Metabolism Division, Wyeth-Ayerst Research, Princeton, New Jersey reported the initial preclinical PK and metabolic disposition studies of VEN (WY-45,030) including its enantiomers and ODV (WY-45,233) in mouse, rat, dog, and rhesus monkey while VEN and ODV were still under development. The analysis of plasma and urine obtained after the animals received a pure aqueous solution of VEN and ODV, and not any kind of dosage formulation, intravenously (*i.v.*) and/or intragastrically (*i.g.*) was conducted by high-performance liquid chromatography (HPLC) method with UV detection. The Wyeth-Ayerst Research also reported an HPLC method for simultaneous estimation of VEN and ODV in rat, dog, and mouse plasma^18,21–23^. In the last 30 years, after its approval for clinical use, a sizeable number of bioanalytical methods for the quantification of VEN and ODV in human plasma and urine have been reported in the literature, but the same does not hold for preclinical animal models. After a comprehensive review of available literature to date, the following bioanalytical methods for carrying out preclinical PK studies of VEN and ODV in animals were found and almost all of these reported administering aqueous solutions of VEN and ODV in its pure form through an oral, *i.v.* or *i.g.* route. da Fonseca and Bonato reported an in vitro enantioselective biotransformation study of VEN to its metabolites in liver microsomal preparations of male Wistar rats using chiral HPLC separation with UV detection^24^. Zhang et al*.* developed the HPLC method with mass spectrometric detection (LC–MS) for the *pK* studies of VEN solid dispersions in male Wistar rats^25^. The tandem mass spectrometry (MS/MS), having higher specificity and improvements like high signal-to-noise ratio, accuracy, and reproducibility, coupled with HPLC has become an apt and more effective tool for extremely low detection limits and is considerably applied to PK studies at present^26^. Many papers have reported HPLC and ultra-high performance liquid chromatography (UHPLC/UPLC) tandem mass spectrometric methods (LC–MS/MS) for the quantification of VEN and ODV in rat plasma. Shah et al*.* (2009) and Ahmad et al*.* have reported liquid–liquid extraction (LLE) and solid-phase extraction (SPE) based LC–MS/MS methods respectively for the simultaneous estimation of VEN and ODV in rat plasma^27,28^. Aryal et al*.* has successfully utilized an LC–MS/MS method to investigate the PK parameters of VEN and ODV in mice plasma and brain dialysate after an *i.v*. and *i.g* administration of the drug^29^. In another study da Fonseca and Bonato reported the development of a more selective and sensitive technique like the LC–MS/MS method for assessing the kinetic disposition of VEN, ODV, and *N*-desmethylvenlafaxine (NDV) in rat plasma after the oral administration of VEN^30^, since the quantification limit attained by previously developed HPLC–UV method^24^ was appropriate for in vitro biotransformation studies only. An LC–MS/MS method was used by Zhang et al*.* to determine the concentration of phenolic esters of ODV, and, Liu et al*.* to estimate synthetic prodrugs of ODV in plasma, brain, and hypothalamus of male Wistar rats, along with PK parameters in beagle dogs in both experimentations after administering the aqueous drug solutions *i.g.* and orally to animals^31,32^. The validation of a UPLC-MS/ESI method developed for simultaneous determination of VEN and ODV in rat plasma and its use in PK studies in male Wistar rats, after administering the VEN solution orally, has been carried out by Dubey et al.^33^. Pan et al*.* claimed to have developed a fully validated UPLC-MS/MS method for simultaneous estimation of methadone, fluoxetine, VEN, and their metabolites in spiked rat plasma for drug interaction study and additionally applied it to PK studies as well^34^. An effective UHPLC-MS/MS method for the simultaneous quantification of VEN and its five metabolites in rat plasma has been reported by Gu et al. and applied to the PK study of VEN orally administered to rats^35^. Chen et al*.* has reported a UPLC-MS/MS method to investigate the underlying mechanism of the effect of vonoprazan on VEN in rat liver microsomes along with its impact on the PK profile of VEN and ODV in male Sprague–Dawley rats after oral administration of drugs^36^. An SPE based gas chromatography mass spectrometric (GC–MS) method for the estimation of venlafaxine in rat plasma and its application to assess PK interaction between VEN and fluoxetine in Sprague–Dawley rats has been reported by Song et al.^37^. One spectrofluorometric method for the estimation of VEN in spiked rat plasma reported by Shahnawaz et al. was also found in literature^38^.

Nerkar and Gattani in two separate studies have reported a comparative PK profile of *i.v.*, oral solution and buccal mucoadhesive gel formulations (Cress seed and Linseed based) containing VEN in New Zealand white rabbits using an HPLC–UV method^39,40^. In another PK study reported by Peng et al., an HPLC method has been employed for the determination of VEN in rabbits after injecting a VEN saline solution and thermosensitive VEN-chitosan hydrogel subcutaneously^41^. Ali et al. has also employed an HPLC–UV method for PK studies of VEN containing oral solution and sustained release hydrogel composites in rabbits^42^. An HPLC–UV method developed and validated in rabbit plasma by Sher et al. has been applied to PK analysis of VEN in albino rabbits after its oral administration^43^.

After reviewing the available literature thoroughly, only two reports of preclinical PK studies of a prototype ER solid oral dosage formulation containing VEN conducted in any animal model were found. The first one is of a VEN ER tablet reported in Rowley et al. United States patent US 20050136109A1 by Wyeth. The patent mentions the PK studies of VEN ER tablets conducted in Beagle dogs, however, no account of the bioanalytical method utilized for estimation of VEN and ODV from dog plasma is stated in the patent publication^44^. The other one is an in vivo PK study of chitosan-carbomer matrix tablets containing VEN conducted by Zhang et al. in Beagle dogs employing an HPLC method^45^.

The present study, to the best of our knowledge, is the first report on preclinical PK studies of ER solid oral dosage formulations of venlafaxine in New Zealand White rabbits. The study involves bioequivalence (BE) assessment of an in-house produced VEN ER tablet and the US FDA reference listed drug (RLD) Efexor XR 37.5 mg employing an SPE based robust, reliable, and validated ultra-high performance LC–MRM–MS/MS method for simultaneous quantitation of VEN and ODV in rabbit plasma.

## Methods

### Chemicals and materials

Working standards of venlafaxine hydrochloride and *O*-desmethylvenlafaxine of 99.83% and 99.82% purity respectively were purchased from Vivan Life Sciences (India). Deuterium labeled venlafaxine-D6 hydrochloride (VEN-D6) of 99.46 atom% D-isotropic enrichment and 99.68% purity and Rac-*O*-desmethylvenlafaxine-D6 succinate hydrate (ODV-D6) of 99.92 atom% d-isotropic enrichment and 99.02% purity was also procured from Vivan Life Sciences (India) and used as an internal standard (IS). Reagent grade ammonium formate and HPLC grade acetonitrile and methanol were obtained from Honeywell Specialty Chemicals (GmbH). Reagent grade orthophosphoric acid and liquor ammonia were acquired from Thermo Fisher Scientific (India). Reagent grade formic acid was obtained from Sigma-Aldrich Chemicals (India). All aqueous solutions and buffers were prepared using ultrapure Milli-Q water. SPE cartridges (Bond *Elut* PLEXA*,* 30 mg/1 cc) were obtained from Agilent (USA). Sodium heparin-containing blood collection tubes, BD VACUTAINER, were purchased from Becton Dickinson (USA). Efexor XR 37.5 mg was purchased from Pfizer Ireland Pharmaceuticals (Ireland).

### Preparation of calibration standards and quality controls

Stock solutions (1 mg mL^−1^) of VEN and ODV were prepared by dissolving accurately weighed standard compounds in methanol. The working solution in the range of 10–5000 ng mL^−1^ was prepared by diluting the stock solution with a diluent (50% methanol in water, v:v). The calibration standard (CS) and quality control (QC) samples were prepared from working solution using diluent and spike-in rabbit plasma (%spiking ~ 5%). The calibration curve (CC) ranged from approximately 0.5 to 250.0 ng mL^−1^ for both analytes. QC samples were prepared at the limit of quantitation (LOQQC), low (LQC), medium (M1QC, MQC), and high (HQC) concentration levels. The nominal concentration of CS and QC samples prepared in rabbit plasma is given in Table 1.

**Table 1:** Nominal concentration of calibration standard and quality control samples.

Samples	ID	Nominal conc. (ng mL^−1^)	
		VEN	ODV
CSs	STD-A	0.503	0.493
	STD-B	1.378	1.350
	STD-C	6.891	6.749
	STD-D	19.689	19.283
	STD-E	49.222	48.208
	STD-F	98.444	96.416
	STD-G	196.889	192.833
	STD-H	255.700	250.432
QCs	LOQQC	0.504	0.494
	LQC	1.392	1.363
	M1QC	49.222	48.208
	MQC	98.444	96.419
	HQC	196.889	192.883

### Sample preparation

For sample preparation, the SPE method was developed for the extraction of VEN and ODV from rabbit plasma. Frozen samples were allowed to thaw at room temperature before vortex-mixing to homogenize the contents. To an 80 µL aliquot of rabbit plasma in a polypropylene tube, 50 µL of IS dilution (containing ~ 25.0 ng mL^−1^ of VEN-D6 and ODV-D6) followed by 400 µL of pretreatment solution (10% orthophosphoric acid in the water, v:v) was added and vortexed. The samples were transferred to SPE cartridges (preconditioned with 0.5 mL of methanol followed by 0.5 mL of Milli-Q water and centrifuged at 2000 rcf for a minute after each addition) and centrifuged at the rcf same as that of preconditioning for one minute. The cartridges were washed with 1 mL of washing solution (5% liquor ammonia in water, v:v) and Milli-Q water by running a centrifuge at 2000 rcf for a minute after each addition. The samples were eluted with 1 mL of methanol by centrifuging at 2000 rcf for a minute and dried under nitrogen steam at 50 ± 4 °C and about 20 ± 5 psi. The dried sample residues were reconstituted in 300 µL of mobile phase, vortexed and an aliquot of the resulted sample was injected onto the LC–MS/MS system for analysis.

### Liquid chromatography (LC)

A Shimadzu Nexera X2 UHPLC system consisting of two Nexera X2 LC-30AD pumps, a Nexera X2 SIL-30AC MP auto sampler, an online DGU-20ASR degassing unit, and a CTO-20AC Prominence HPLC column oven was used for LC. Chromatographic separation was achieved via isocratic elution of mobile phase (60% methanol in 2 mM ammonium formate buffer (v:v) + 0.1% formic acid L^−1^) at a flow rate of 0.300 mL min^−1^ on an ACQUITY UPLC BEH C_18_ column (2.1 × 100 mm, 1.7 µm) with a total run time of 3 min. An injection volume of 5 µL was used for each analysis. The retention time for the analytes and their respective stable isotopically labeled internal standards (SIL-IS) is given in Table 2. The auto sampler and column oven temperature were set at 10 ± 1.0 °C and 40 ± 1.0 °C respectively. The composition of the rinsing solution used was 90% acetonitrile in water (v:v).

**Table 2:** Retention time for the analytes and their SIL-IS.

Analytes/SIL-IS	Retention time (min)
VEN	1.1–1.4
VEN-D6	1.1–1.4
ODV	0.75–1.1
ODV-D6	0.75–1.1

### Tandem mass spectrometry (MS/MS)

The eluted samples from LC were subsequently analyzed using an AB SCIEX triple quadrupole (QqQ) API 4000 LC/MS/MS system operated in multiple-reaction monitoring (MRM) mode. The ion source used was TURBO ION SPRAY, an electrospray ionization (ESI) probe, in positive polarity. Two MRM transitions, Q1 (precursor ion) and Q3 (product ion), with a dwell time of 200 ms for simultaneous quantitation and identification of the analytes (VEN and ODV) and their SIL-IS (VEN-D6 and ODV-D6) based on mass-to-charge ratio (*m/z*) calculation and compound dependent parameters were optimized.

### Data processing and statistical evaluation

LC/MS/MS data was processed using the ANALYST software version 1.6.3 from AB SCIEX. The CC was generated through a weighted (X^−1^, X^−2^, and none, where X = concentration) linear regression, and values for slope, intercept, and correlation coefficient (R) were obtained. The concentration of VEN and ODV was determined by plotting the peak area ratio of analyte/IS based on CC. Mean, standard deviation (SD), precision (%CV), accuracy (%Nominal) were calculated using MS Excel software.

### Validation

The method developed was validated in line with US FDA Guidance for Industry Bioanalytical Method Validation 2001^46^, US FDA Bioanalytical Method Validation Guidance for Industry 2018^47^, and US FDA ICH Harmonized Guideline M10 Bioanalytical Method Validation (2018)^48^. The validation was done with regard to the linearity of CC, sensitivity at LOQ, selectivity, matrix effect, carryover, precision and accuracy (PA), recovery, and incurred sample reanalysis (ISR) as per the FDA recommendations and acceptance criteria for the bioanalytical method validation.

### Manufacture and dissolution testing of ER tablets

Direct compression method was used to produce the VEN ER tablets of 140.0 mg weight and hardness of around 4–5 kPa using a rotary tablet press with 7.25 mm round concave tooling. In vitro dissolution testing was performed according to the USP 35 “Dissolution procedure” < 711 > using USP apparatus ӀӀ (paddle) method at 50 rpm in 900 mL dissolution medium at 37 °C. The dissolution samples taken were analyzed spectrophotometrically at 225 nm using a 5 mm path length cuvette.

### Preclinical PK studies of VEN and ODV in rabbits

The bioanalytical method developed was used for simultaneous quantitation of VEN and ODV in rabbit plasma and applied for carrying out preclinical PK study in New Zealand White rabbits. The animal experiments were approved by the Institutional Animal Ethics Committee (IAEC) of the Department of Pharmaceutical Sciences, University of Kashmir [Ref. No. F(IAEC-Approval)KU/2017/09]. The animal experiments were conducted as per the relevant Indian Laws [The Breeding and Experiments on Animals (Control and Supervision) Rules 1998, Amendment Rules 2001, and Amendment Rules 2006], and ARRIVE guidelines. Four pairs (male/female) of healthy rabbits, each weighing 3–4 kg, were provided by Government Rabbit Farm, Wussan (Pattan), Kashmir. The rabbits were divided into a reference group and a test group. Each of the 4 rabbits in the reference group received a capsule of Efexor XR 37.5 mg orally and in the same manner rabbits in the test group received an in-house produced VEN ER tablet. After administration, 2 mL of blood was collected from the marginal ear vein at 0, 1, 2, 3, 4, 6, 8, 12, 24, and 36 h in sodium heparin containing BD VACUTAINER. The cell free plasma from anticoagulated blood was obtained by centrifuging the blood samples at 3000 rcf and 17 °C for 15 min as per the WHO Use of Anticoagulants in Diagnostic Laboratory Investigations 2002 Guidelines^49^. The resulting plasma samples were frozen at − 40 °C until further analysis.

## Results and discussion

### Method development

During method development, SPE was found to be more selective for removing the interferences like dissolved salts (electrolytes) and proteins from the plasma. The reliability of the method was ascertained from the results of the recovery experiment delivering excellent reproducibility. The requirement of an 80 µL aliquot of rabbit plasma also ensured less wasted sample. A non-ionic and non-polar base load method for primary extraction of analytes having Log P > 1.5 and pK_a_ of 6–10 was selected, and SPE cartridges containing hydrophilic non-polar styrene divinylbenzene polymeric sorbent were used for this purpose. The sorbent conditioning was achieved with 100% methanol followed by equilibrating with 100% water. The acidic treatment of plasma with orthophosphoric acid helped in protein precipitation and made plasma ready for extraction loading. During loading the gradient of polarity on the hydrophilic sorbent surface allowed phase transfer of analytes into the more hydrophobic polymer core for retention prior to washing and subsequent elution. The large endogenous matrix components did not reach the core due to their inability to bind to the polymeric surface. The washing treatment with liquor ammonia removed the interferences without leaching of analytes. The clean extract with high recovery was obtained by eluting the plasma sample with 100% methanol.

The focus during LC was on obtaining sharp peaks with high resolution for the analytes under investigation and attaining high efficiency coupled with maximum MS/MS sensitivity. The chromatographic separation was realized isocratically using reversed-phase LC (RPLC). According to the fundamental resolution equation for isocratic separations, the resolution depends on column efficiency, which in itself is influenced by the particle size of the stationary phase. Since the smaller particles (sub-2-µm) provide higher chromatographic resolution, the UPLC BEH C_18_ column was selected for RPLC. The stationary bonded phase of this column contains highly efficient 1.7 µM ethylene bridged ethyl-siloxane/silica hybrid (BEH) particles bonded with hydrophobic trifunctional C_18_ ligands utilizing proprietary endcapping processes. The bonded phase has a ligand density of 3.1 µMol M^−2^ and a carbon load of 18%. Since retention in RPLC is primarily related to solute hydrophobicity, for the molecule more hydrophobic to be separated, the lesser hydrophobic ligand ought to be used. VEN and ODV being highly hydrophilic molecules, strongly hydrophobic trifunctional C_18_ ligands were able to provide sufficient binding for the desired separation. The appropriate adjustment of the polarity of the mobile phase is equally important as the selection of the non-polar column for the separation of solute molecules in RPLC. The predominantly polar solvent methanol was optimized as the mobile phase and the adequate buffering capacity of the mobile phase was maintained with ammonium formate (pK_a_ = 3.75 and buffer range of 3.76–5.76). Since the flow rate of the mobile phase plays an important role in the resolution of small molecules in reversed phase separations, attuning it to 0.300 mL/min helped in obtaining a high resolution for the analytes.

The two main targets taken into consideration while optimizing MS/MS conditions were achieving adequate sensitivity and selectivity. Although sample extraction, chromatography development, and MS initial tuning all have an effect on sensitivity and selectivity, MS optimization considerably influences the sensitivity of the method. During the MS initial tuning, the first step was determining the ionization of analytes. The ionization source/gas parameters (Table 3) such as gas flow and ionization voltage were tuned to the analytes making optimum ionization conditions for the analyte and subsequently sensitivity. It was during this MS initial tuning, the positive ionization mode was determined for carrying out MS/MS analysis. The appropriate ionization mode and detection polarity was selected based on analyte polarity and LC operating conditions. The major improvement the use of ESI offers is the formation of protonated or deprotonated molecules with little fragmentation, which is ideal for the selection of precursor ions including maximizing sensitivity.

**Table 3:** Parameter optimization for electrospray ionization (ESI) probe.

Parameter	Values
Gas 1—Ion Spray nebulizer gas	60 psig
Gas 2—TURBO ION SPRAY heater gas	40 psig
Temperature	500 °C
Curtain gas	30 psig
Ion Spray voltage	5500 V
Collision gas	6 psig
Interface heater	ON

**Figure 2:**
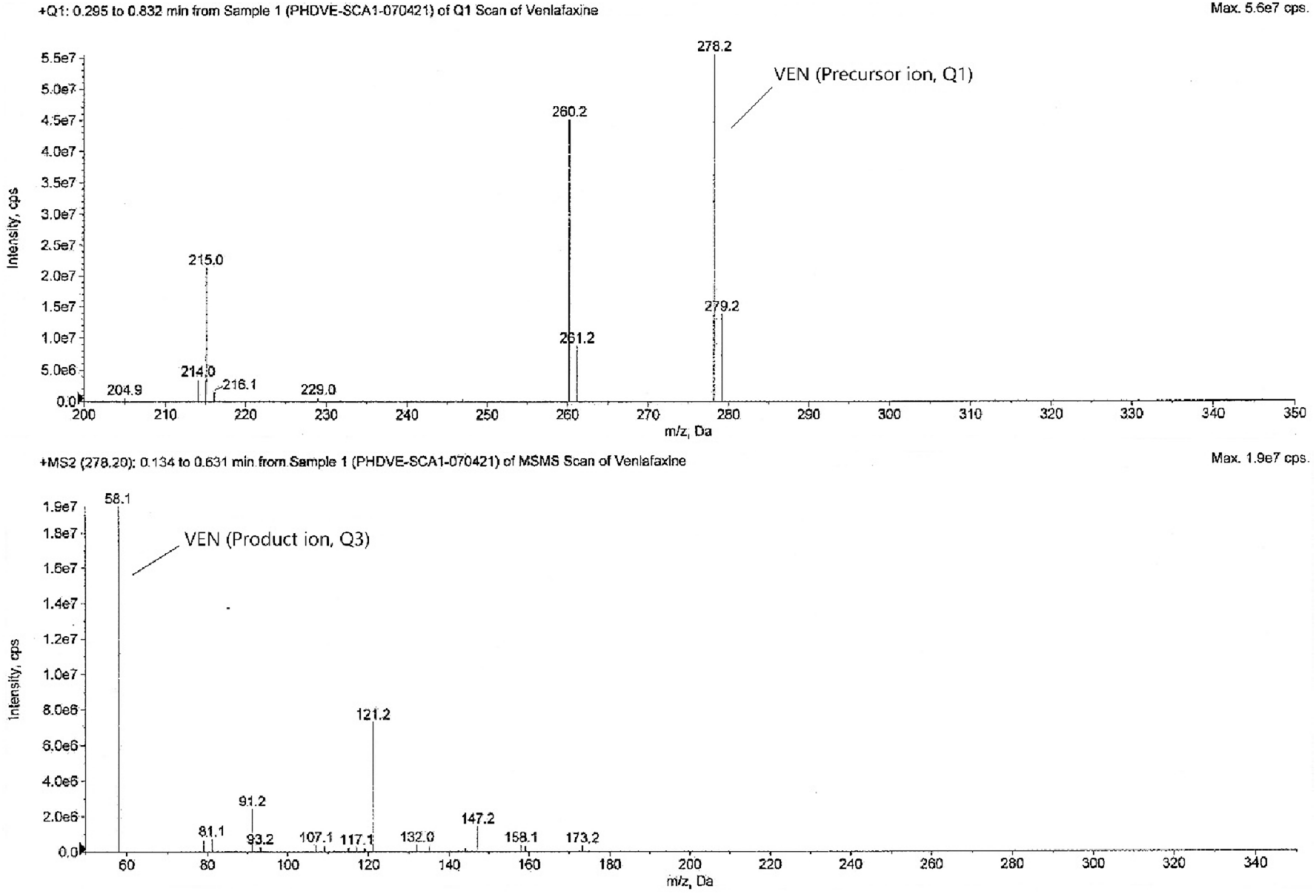
MRM mass spectra and fragmentation of venlafaxine.

**Figure 3:**
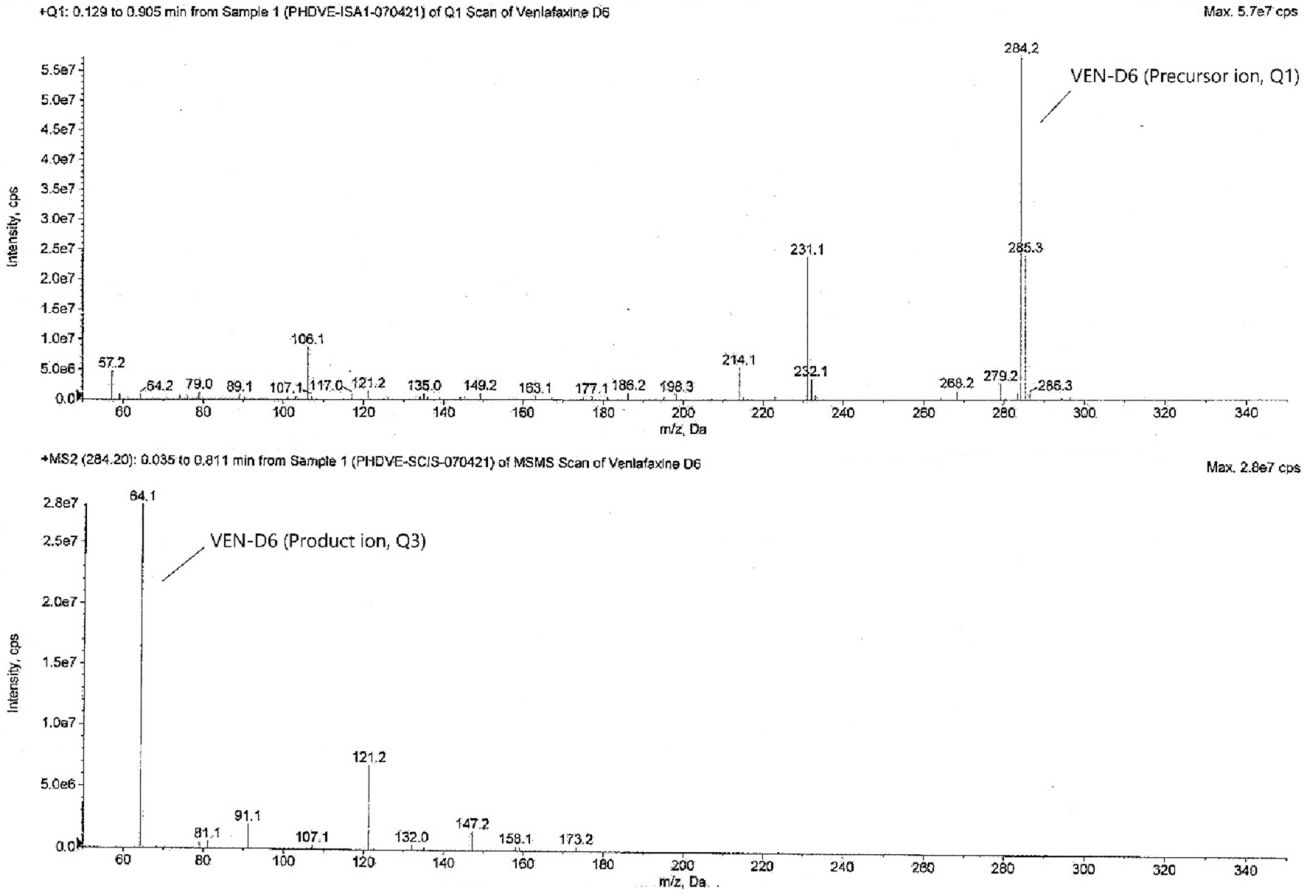
MRM mass spectra and fragmentation of venlafaxine-D6.

**Figure 4:**
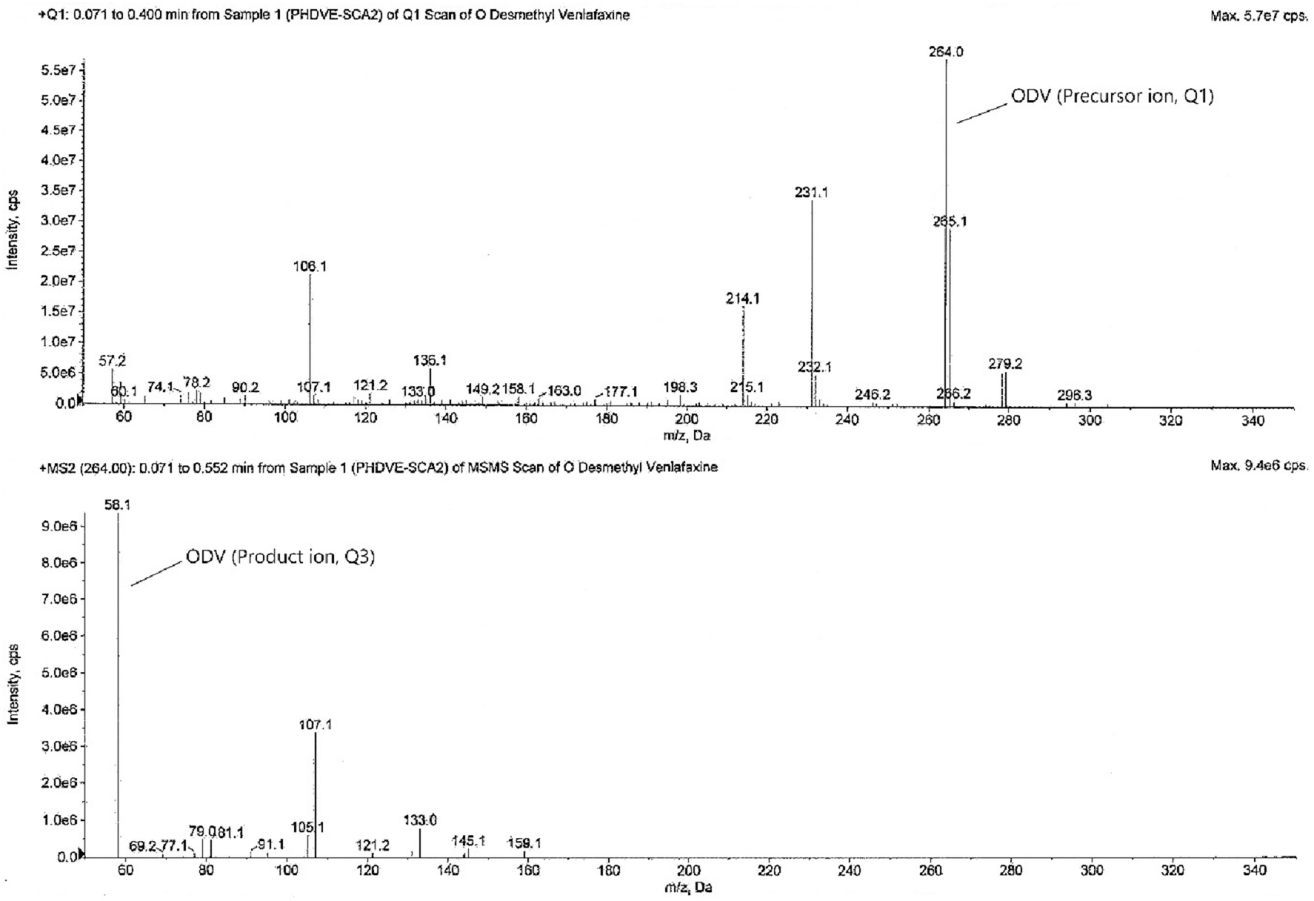
MRM mass spectra and fragmentation of *O*-desmethylvenlafaxine.

**Figure 5:**
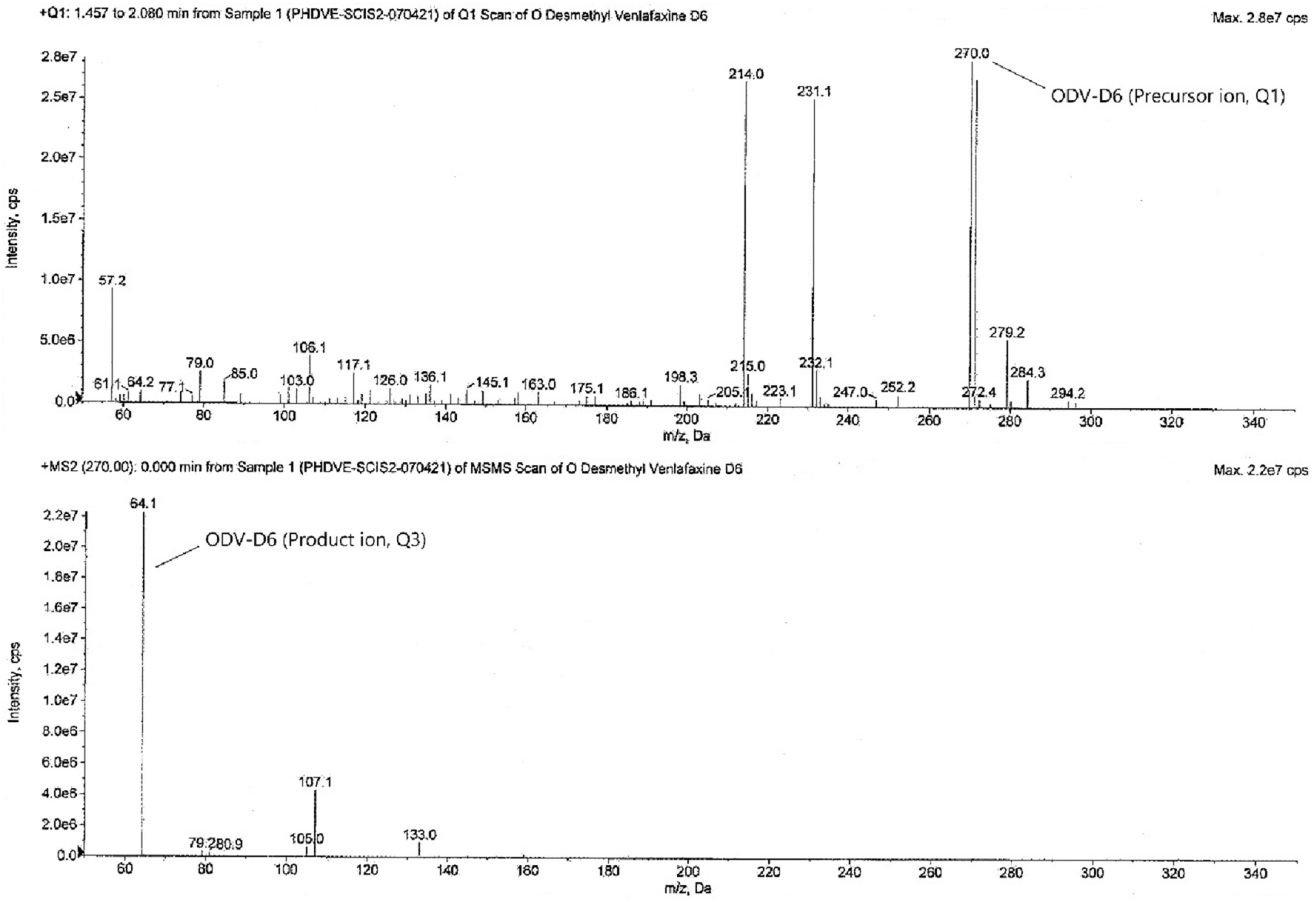
MRM mass spectra and fragmentation of *O*-desmethylvenlafaxine-D6.

The tandem mass spectrometry is principally based upon precursor ion selection (Q1), its fragmentation mostly by collision-induced dissociation (CID), and the *m/z* measurement of the product ions (Q3) formed. The screening of two to three MRM transitions for each analyte, in the beginning, gives the confidence that the correct analyte is being monitored and this selectivity helps in the later stages of method development when actual biological samples are analyzed. The optimized MS parameters for each compound are given in Table 4. The analytes were detected by collision-induced MS/MS employing MRM and mass transition ion-pair (precursor-product ion transitions) for VEN, VEN-D6, ODV, and ODV-D6 was selected as *m/z* 278.2 → 58.1, *m/z* 284.2 → 64.1, *m/z* 264.0 → 58.1, and *m/z* 270.0 → 64.1 respectively. The MRM mass spectra and fragmentation of the analytes and SIL-ISs are given in Figs. 2, 3, 4, and 5.

**Table 4:** Optimized MS parameters for MRM transitions of each compound.

Compound	MRM		Compound parameters			
	Q1 (amu)	Q3 (amu)	DP (V)	EP (V)	CE (V)	CXP (V)
VEN	278.2	58.1	40	10	35	6
VEN-D6	284.2	64.1	40	10	35	6
ODV	264.0	58.1	40	10	35	6
ODV-D6	270.0	64.1	40	10	35	6

The MRM-MS spectrometry based bioanalytical method development coupled with the quantitation accuracy provided by the use of SIL-IS improved the sensitivity and selectivity through variability controlling in the quantitation of the analytes, and, also helped in simultaneously monitoring the masses of the light and heavy precursor ions. The MRM distinguishes compounds based on mass, thus eliminating the requirement to separate every single component present in the sample by the chromatographic method used. Therefore, the MRM-MS spectrometry on the QqQ MS platform provided the speed and accuracy to detect multiple transitions (Q1/Q3 pairs) allowing fast LC analysis and reduced sample preparation time. Moreover, the MRM-MS based simultaneous quantitation of VEN and its active metabolite enabled in development of a high throughput bioanalytical method capable to inject both the forms in the same run, thus harmonizing for run-to-run variations in sensitivity, sample-specific ion suppression, and, deviations in retention time. The use of MRM with SIL-IS, which is also credited as the gold standard of MS quantitation, offered consistency in the precision and the reproducibility of the bioanalytical method developed. The reproducibility of the MRM-MS spectrometry method developed was found to be reliable as demonstrated through ISR evaluation results. The ISR data showed that the reanalyzed quantitation value of VEN and ODV was ± 20% of the mean in 93.35% and 86.96% of the total number of reanalysis samples respectively.

### Method validation

#### Linearity of CC

All blank, CS, and QC samples were prepared in the plasma obtained from the same rabbits as the study samples. Three batches of spiked plasma samples containing eight non-zero CS levels including a LOQ and covering the quantitation range were run for linearity of CC. Every run also included 2 replicates of each LQC, M1QC, MQC, and HQC. All non-zero CS levels were found to be ± 15% of the nominal concentrations as per the FDA acceptance criteria. The concentration–response relationship was found to be fit with the simplest regression model and showed the linear character of CC. The average CC parameters for each analyte are presented in Table 5.

**Table 5:** Average *CC* parameters for VEN and ODV in rabbit plasma.

Average calibration curve parameters				
Analyte	CC run	Slope	Intercept	R
VEN	I	0.0427	− 0.000968	0.9995
	II	0.0433	− 0.00114	0.9996
	III	0.0422	0.00068	0.9997
ODV	I	0.0392	0.00437	0.9995
	II	0.0407	0.00431	0.9991
	III	0.0385	0.00376	0.9994

#### Sensitivity at LOQ

**Figure 6:**
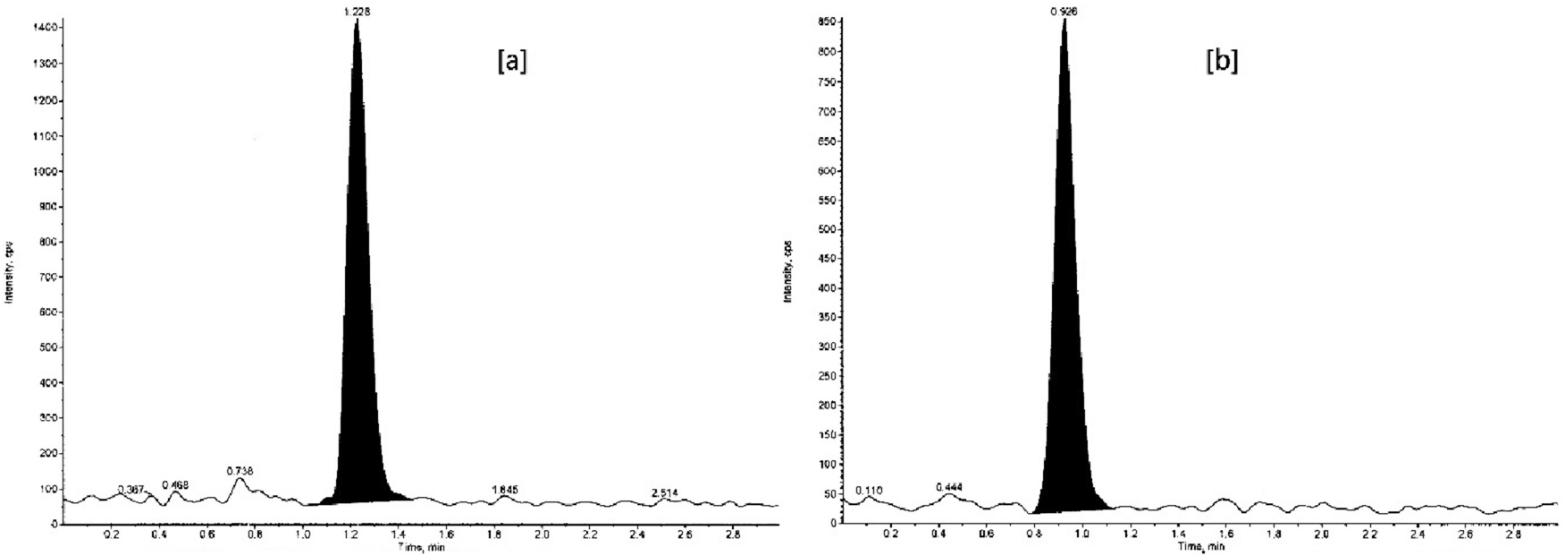
Representative chromatogram of LOQQC of (**a**) venlafaxine and (**b**) *O*-desmethylvenlafaxine.

The lowest non-zero CS level on the CC defines the LOQ. FDA acceptance criteria require LOQ to be ≥ 5 times the analyte response of the zero CS level (blank), with an accuracy of ± 20% of the nominal concentration and reproducible with a precision of ± 20% (%CV). Sensitivity at LOQ was done as a part of PA assessment for the CC range and was determined by running the LOQQC (Fig. 6) in 6 replicates in three within-day PA batches. The results given in Table 6 show that LOQ was quantitatively determined within the acceptable precision and accuracy criteria.

**Table 6:** Sensitivity at LOQ for each analyte in rabbit plasma.

Sensitivity at LOQ				
Analyte	LOQQC run (within-day)	SD (±)	Precision (%CV)	Accuracy (%Nominal)
VEN	PA batch—I	0.10805	19.15	111.97
	PA batch—II	0.10248	18.29	111.14
	PA batch—III	0.02192	4.70	92.56
ODV	PA batch—I	0.07833	15.30	103.61
	PA batch—II	0.08118	16.38	100.30
	PA batch—III	0.02701	6.13	89.24

#### Selectivity

**Figure 7:**
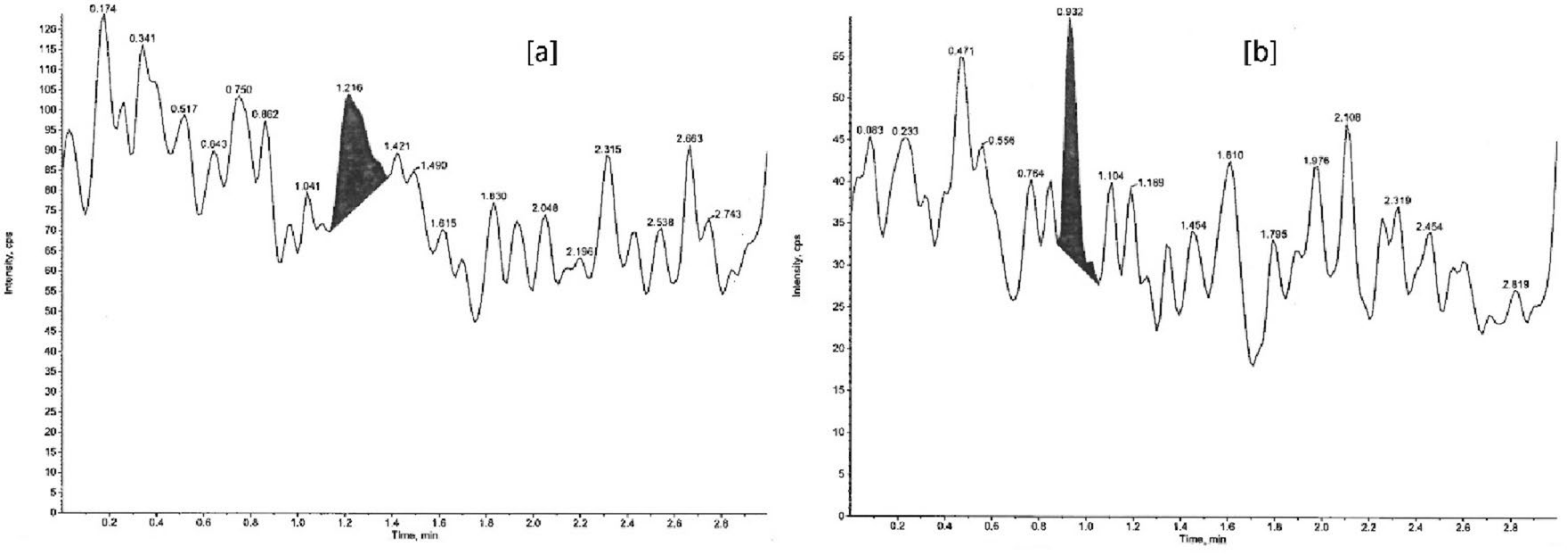
Representative chromatogram of the double blank of (**a**) venlafaxine and (**b**) *O*-desmethylvenlafaxine.

FDA guidelines ask for carrying out selectivity during validation to lessen or avoid interference from potential interfering endogenous components of matrix and verify the substance being measured is the intended analyte. Selectivity was demonstrated by analyzing 10 blank samples of rabbit plasma (double blank), and, the peak area response at retention times of analytes (VEN and ODV) and ISs (VEN-D6 and ODV-D6) in both blank matrix and extracted LOQ was measured (Fig. 7). The extraction method used was the same as mentioned in sample preparation “Sample preparation”. The %area of the blank matrix to mean peak area response of analyte in extracted LOQ for VEN and ODV was 1.25–4.22% and 1.07–9.22% respectively. For both ISs, VEN-D6 and ODV-D6, the %area of the blank matrix to mean peak area response of IS in extracted LOQ was 0.00–0.02%. The results were well within FDA acceptance criteria and showed blanks to be free of interference for the analyte (< 20% peak area response of analyte in blank compared to extracted LOQ samples) and the IS response in blank didn’t exceed 5% of the average IS responses of CSs and QCs.

#### Matrix effect

**Figure 8:**
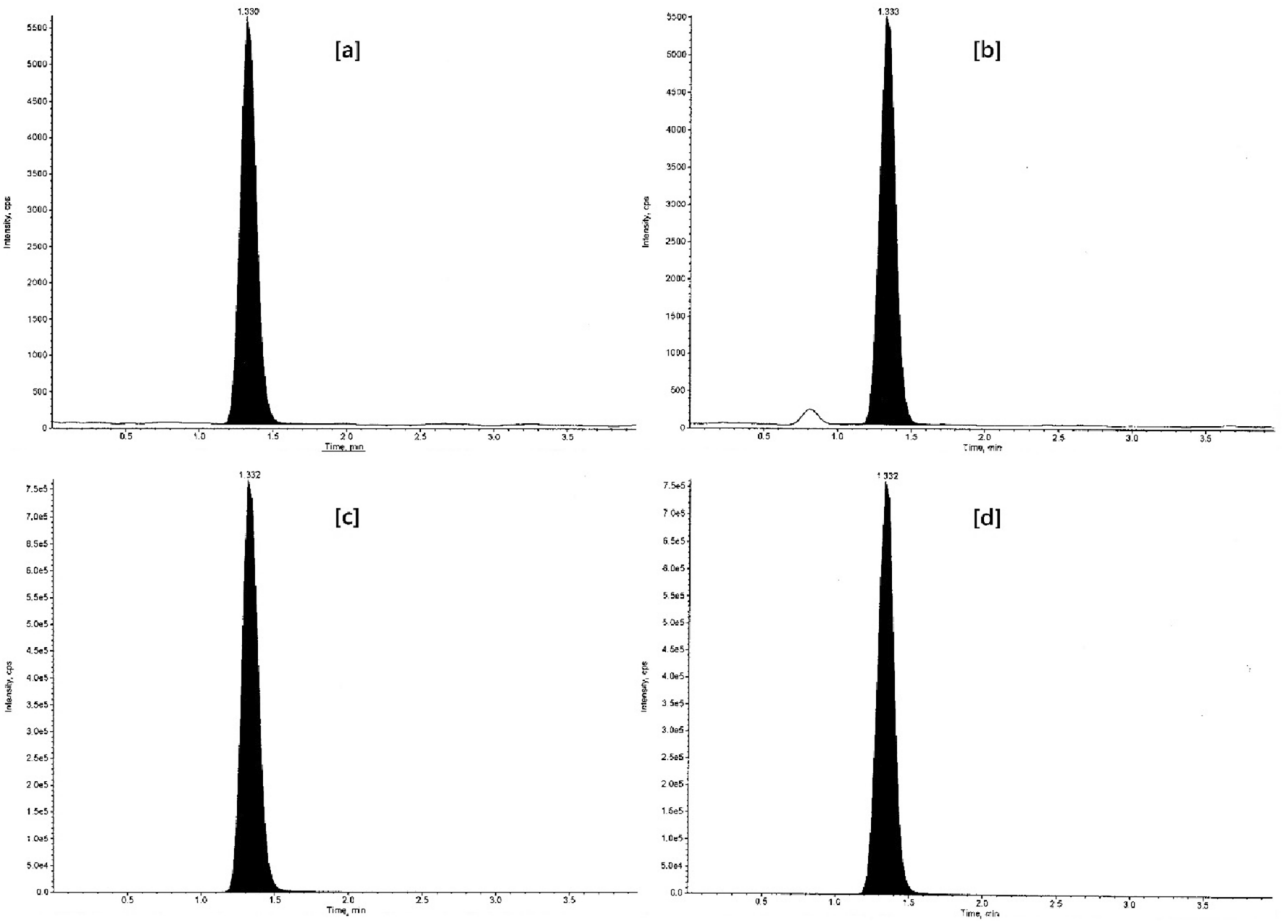
Representative chromatograms of venlafaxine at (**a**) LQC (spiked), (**b**) LQC (pure standard solution), (**c**) HQC (spiked), and (**d**) HQC (pure standard solution) level.

**Figure 9:**
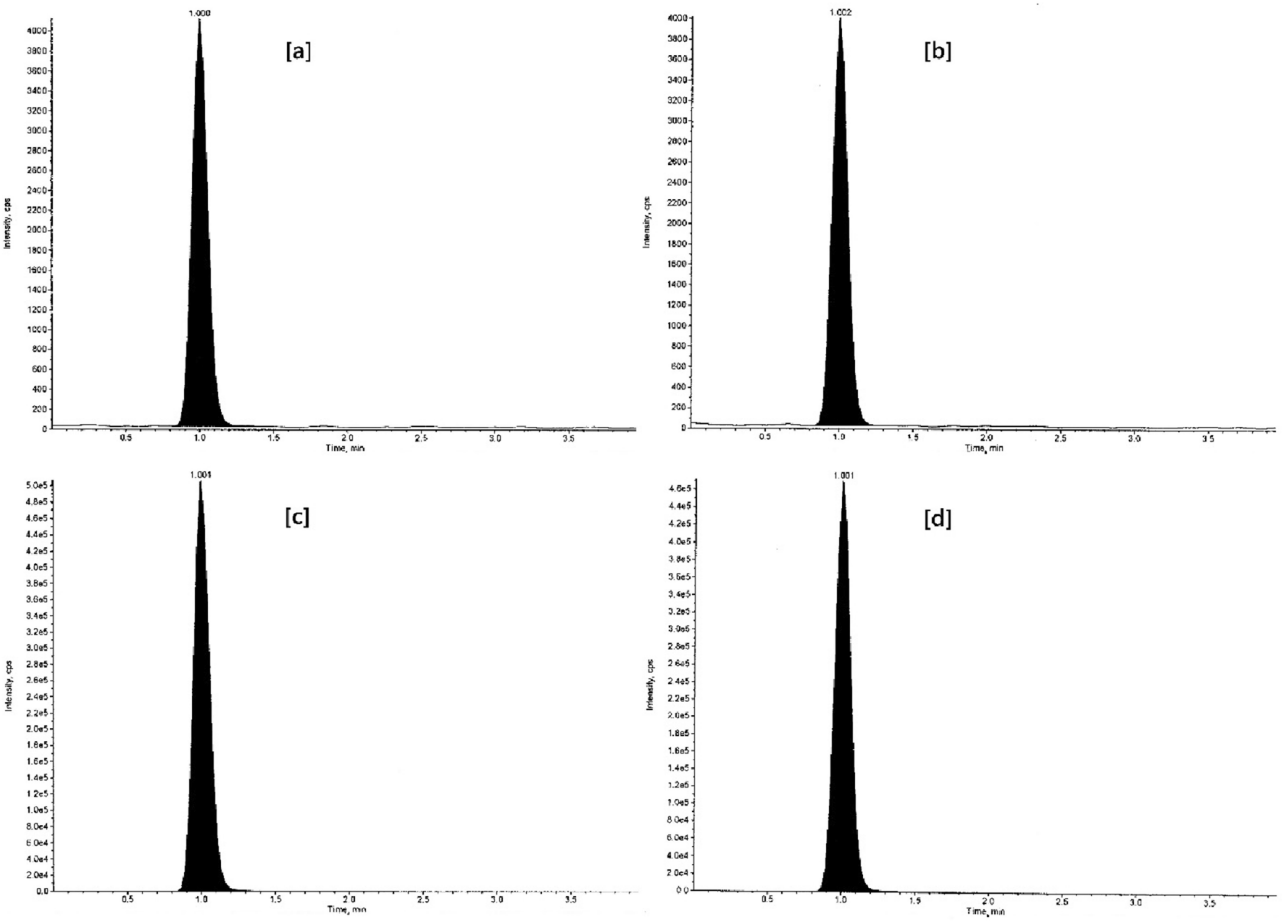
Representative chromatograms of *O*-desmethylvenlafaxine at (**a**) LQC (spiked), (**b**) LQC (pure standard solution), (**c**) HQC (spiked), and (**d**) HQC (pure standard solution) level.

The FDA recommendations require the matrix samples to be tested for matrix effect so that the precision, selectivity, and sensitivity are not compromised throughout the application of an LC–MS and LC–MS/MS method. The matrix effect is observed as the shift in an analyte response normally due to the presence of an unknown and interfering component/s in the sample matrix. The matrix effect, quantitatively measured in terms of matrix factor (MF), was estimated as the ratio of the analyte peak response obtained at LQC and HQC level (Figs. 8, 9) in the presence (extracted and spiked post-extraction) and absence of matrix ions (pure standard solution). Furthermore, the IS-normalized-MF or Absolute-MF was calculated as the ratio of MF of an analyte to the MF of an IS. Since the use of SIL-IS reduces the effective IS-normalized-MF variability, it is not necessary to determine MF or IS-normalized-MF in 6 lots of independent matrix samples^50^. The variability in the MFs, as determined by the %CV (Table 7), was found to be well within the FDA ICH Harmonized guideline acceptance criteria (less than 15%).

**Table 7:** Matrix effect data for VEN and ODV in three different lots of rabbit plasma.

Analyte	VEN		
IS	VEN-D6		
QC level	VEN-MF	VEN-D6-MF	IS-normalized-MF
LQC	0.9853	0.9645	1.0215
	0.9918	0.9893	1.0025
	0.8935	0.8380	1.0662
	Mean		1.03006
	SD (±)		0.03270
	Precision (%CV)		3.17
HQC	0.9943	0.9881	1.0063
	1.0053	0.9737	1.0324
	0.8720	0.8405	1.0374
	Mean		1.02536
	SD (±)		0.01670
	Precision (%CV)		1.63

Analyte	ODV		
IS	ODV-D6		
QC level	ODV-MF	ODV-D6-MF	IS-normalized-MF
LQC	0.9661	0.9339	1.0344
	0.9644	0.9744	0.9897
	0.6740	0.8380	0.9606
	Mean		0.99492
	SD (±)		0.03717
	Precision (%CV)		3.74
HQC	0.9333	0.9517	0.9806
	0.9503	0.9570	0.9930
	0.7090	0.7236	0.9798
	Mean		0.98447
	SD (±)		0.00740
	Precision (%CV)		0.75

#### Carryover

**Figure 10:**
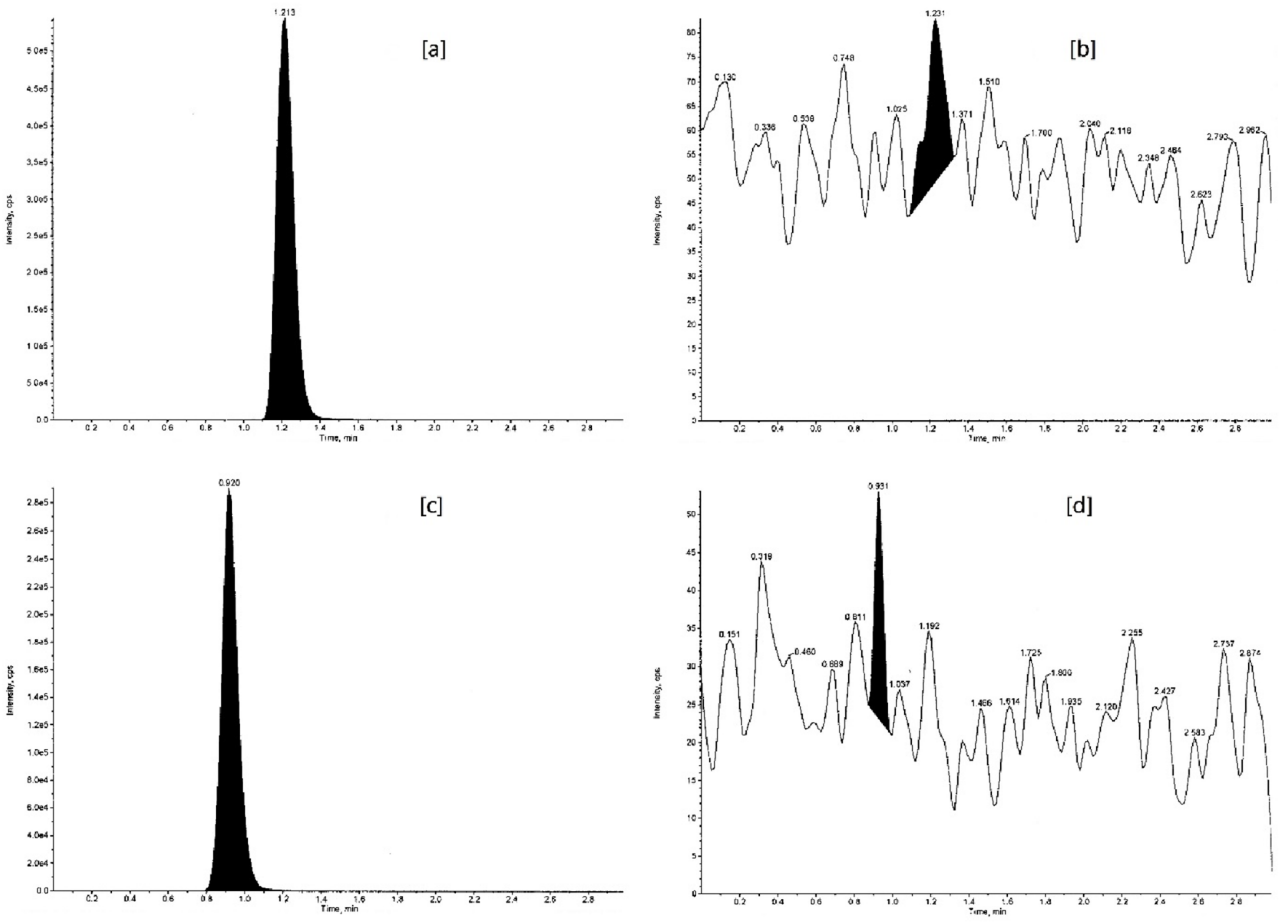
Representative Carryover chromatograms of (**a**) venlafaxine HQC, (**b**) subsequent venlafaxine double blank, (**c**) *O*-desmethylvenlafaxine HQC, and (**d**) subsequent *O*-desmethylvenlafaxine double blank.

In an analytical method, carryover between the samples can occur due to the influx of an analyte in a sample from the preceding one. FDA guidelines demand carryover should not exceed 20% of LOQ and urge riddance of any carryover as well as monitoring its impact, if any, on the quantitation of study samples. Carryover was monitored by injecting 3 blank matrix samples (double blank) subsequently after the high CS levels (HQC). The %area of 3 blank matrix samples to mean peak area response of analyte in extracted LOQ for VEN and ODV was 5.17%, 3.11%, 2.43% and 2.72%, 2.26%, 5.98% respectively. Similarly, for VEN-D6 and ODV-D6, the %area of the blank matrix to mean peak area response of IS in extracted LOQ remained in the range of 0.00 to 0.02%. The results exhibit negligible carryover with no enhancement in the peak area response at retention times of analytes and ISs ensuring the carryover didn’t affect the PA of the method developed (Fig. 10).

#### Precision and accuracy

Method validation through weighing PA is essential as per the FDA guidelines to establish the method qualification for analysis of study samples and involves evaluating 4 *QC* levels (LOQQC*,* LQC, MQC, and HQC) across the quantitation range. Between batch (inter-day) and within batch (intra-day) PA was determined by running VEN and ODV QC replicates against the CC on different days (n = 18) and same day (n = 6) respectively. Freshly prepared CSs and QCs were used for each run. A minimum of 3 independent PA runs were carried out along with a CC included in each run. Based on the performance of QCs in all between batch and within batch PA runs, it is evident from the results given in Table 8 that PA of the method is well within the FDA acceptance criteria (precision and accuracy: ± 15% of nominal concentrations of QCs except for ± 20% at LOQQC).

**Table 8:** Between batch and within batch *PA* results for four *QC* levels of each analyte in rabbit plasma.

Analyte	VEN				ODV			
QC levels	LOQQC	LQC	MQC	HQC	LOQQC	LQC	MQC	HQC
Nominal conc. (ng mL^−1^)	0.504	1.392	98.444	196.889	0.494	1.363	96.416	192.833
**Between batch PA**								
Mean conc. (ng mL^−1^*,* n = 18)	0.530	1.368	105.538	208.041	0.482	1.332	102.478	206.946
SD (±)	0.009	0.090	8.974	5.397	0.070	0.096	5.633	6.929
Precision (%CV)	17.71	6.64	8.50	2.59	14.55	7.23	5.50	3.35
Accuracy (%Nominal)	105.22	98.32	107.21	105.66	97.72	97.77	106.29	107.32
**Within batch PA—I**								
Mean conc. (ng mL^−1^*,* n = 6)	0.564	1.370	105.921	208.395	0.511	1.317	102.381	207.529
SD (±)	0.108	0.111	11.781	6.595	0.078	0.114	7.061	9.499
Precision (%CV)	19.15	8.12	11.12	3.16	15.30	8.66	6.90	4.58
Accuracy (%Nominal)	111.97	98.43	107.60	105.84	103.61	96.66	106.19	107.62
**Within batch PA—II**								
Mean conc. (ng mL^−1^*,* n = 6)	0.560	1.369	106.177	208.930	0.495	1.311	102.391	204.891
SD (±)	0.102	0.106	10.380	5.524	0.081	0.106	6.515	6.538
Precision (%CV)	18.29	7.75	9.78	2.64	16.38	8.15	6.36	3.19
Accuracy (%Nominal)	111.14	98.41	107.86	106.12	100.30	96.20	106.20	106.25
**Within batch PA—III**								
Mean conc. (ng mL^−1^*,* n = 6)	0.466	1.365	104.516	206.798	0.440	1.369	102.661	208.420
SD (±)	0.021	0.066	5.034	4.701	0.270	0.068	3.940	4.710
Precision (%CV)	4.70	4.84	4.82	2.27	6.13	4.99	3.84	2.26
Accuracy (%Nominal)	92.56	98.12	106.17	105.03	89.24	100.44	106.48	108.08

#### Recovery

The efficiency and reproducibility of the extraction process used during method development are established by optimizing the recovery of analyte and IS. Although FDA guidelines state that recovery need not be 100%, but maintain that the extent of recovery is consistent and reproducible. The recovery was performed by comparing the analyte peak area (counts) of 6 replicates each of extracted QC samples at LQC, MQC, and HQC with the corresponding extracts of blanks spiked with the analyte post-extraction (representing approximately 100% recovery). The recovery of ISs was estimated in a similar manner using 18 replicates of extracted IS samples. The results of the recovery experiment given in Table 9 confirm the consistency and reproducibility of SPE used in the method development for the simultaneous estimation of VEN, ODV, and their ISs.

**Table 9:** SPE based recovery of VEN, ODV, VEN-D6, and ODV-D6.

Analyte	% Recovery			Mean	SD ( ±)	Precision (%CV)
	LQC	MQC	HQC			
VEN (n = 6/QC)	86.41	89.10	88.05	87.85	1.355	1.54
ODV (n = 6/QC)	72.42	75.91	73.77	74.03	1.760	2.38
**IS**	**% Recovery**					
VEN-D6 (n = 18)	81.52					
ODV-D6 (n = 18)	68.64					

### Development summary of in-house produced ER tablets

A Quality by Design (QbD) approach was used to screen different release controlling polymers to develop an ER tablet formulation of VEN. The optimized formulation produced, using an optimal mixture design of experiments (DOE), for preclinical PK evaluation contained 13.1%, 19.25%, and 36.4% of carbopol 971P, Blanose 7HXF, and Avicel 112 respectively. The appropriate levels of Ligamed MF-2-V and Aerosil 200 were also adjusted to produce a robust formulation. The optimal mixture design helps in customizing the target to meet the specific requirements^51^, which in this case was to match the in vitro drug release with that of the RLD.

The dissolution profile comparison between the optimized ER tablet and RLD in pH 4.5 acetate buffer, pH 5.5 acetate buffer, and pH 6.8 phosphate buffer was carried out as per the US FDA Guidance for Industry, Bioavailability and Bioequivalence Studies for Orally Administered Drug Products—General Considerations (2002)^52^ using similarity factor (f_2_) through model-independent approach^53^. The similarity factor f_2_ values in different dissolution media are provided in Table 10. The dissolution data of the optimized ER tablet and RLD qualified the f_2_ curve similarity test (test vs reference) with an f_2_ value obtained between 50 and 100, which ensured consistency and in vitro equivalence of the two dissolution profile curves.

**Table 10:** f_2_ data of in-house produced VEN ER tablet and Efexor XR.

Strength	Comparison	Dissolution medium	f_2_ result
37.5 mg	Test ER tablet vs Efexor XR	pH 4.5 acetate buffer	65.62
37.5 mg	Test ER tablet vs Efexor XR	pH 5.5 acetate buffer	67.24
37.5 mg	Test ER tablet vs Efexor XR	pH 6.8 phosphate buffer	74.40

### Pharmacokinetic application of the method in rabbits

**Figure 11:**
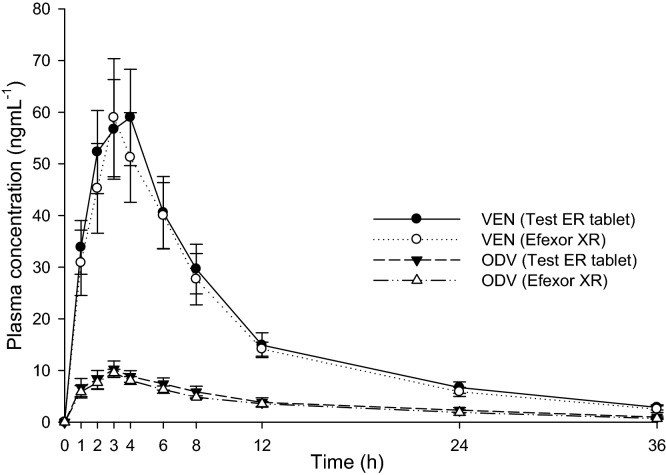
Mean plasma concentration versus time plots after a single oral dose of test ER tablet and Efexor XR 37.5 mg in New Zealand white rabbits.

**Figure 12:**
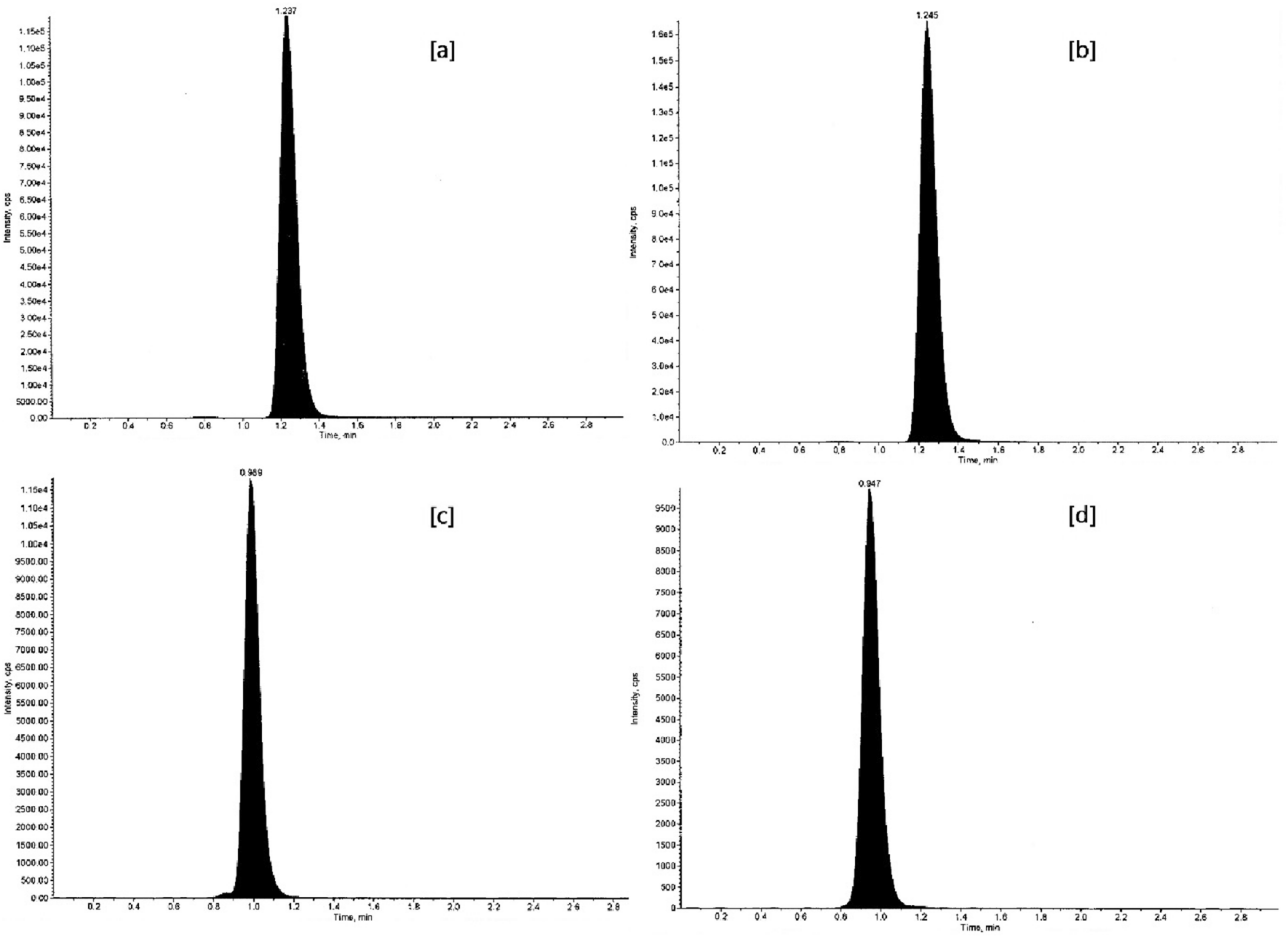
Representative chromatograms of (**a**) venlafaxine C_max_ Test, (**b**) venlafaxine C_max_ Ref, (**c**) *O*-desmethylvenlafaxine C_max_ Test, and (**d**) *O*-desmethylvenlafaxine C_max_ Ref.

The validated LC–MS/MS method was efficiently applied for in tandem quantitation of VEN and ODV in rabbit plasma after oral administration of the optimized ER tablet and RLD as described under heading 2.9. The PK evaluation summarized in Table 11 was carried out by way of noncompartmental analysis (NCA) using WinNonlin 8.1 and the additional statistical computations were performed in SAS 9.4. The BE assessment using bioavailability comparison of key PK parameters between reference and test product was found to be similar. The statistical assurance of bioequivalent similarity given in Table 12 was computed using analysis of variance on log-transformed PK parameters (C_max_, AUClast, and AUCINF__obs_) of reference and test product. The mean plasma concentration *versus* time plots of VEN and ODV are shown in Fig. 11. The plasma concentrations of VEN and ODV remained in the standard quantitation range and above LOQ (0.5 ng mL^−1^) throughout the sampling period (36 h). The representative chromatograms of VEN and ODV C_max_ of reference and test products in given in Fig. 12.

**Table 11:** PK parameters of VEN and free ODV in New Zealand white rabbits (n = 4).

PK parameters	Analyte			
	VEN		ODV	
	Efexor XR	Test ER tablet	Efexor XR	Test ER tablet
T_max_ (h)	3.00	4.00	3.00	3.00
C_max_ (ng/mL)	58.94 ± 13.20	58.99 ± 10.77	9.57 ± 0.75	10.24 ± 1.84
AUClast (h ng/mL)	573.22 ± 99.23	617.85 ± 112.95	116.93 ± 11.63	134.47 ± 30.89
AUCINF_obs (h ng/mL)	608.95 ± 110.35	659.32 ± 120.00	126.90 ± 15.09	148.93 ± 35.41
HL_Lambda_z (h)	9.61 ± 1.34	10.06 ± 0.64	9.72 ± 1.31	10.63 ± 0.49
AUC_%Extrap_obs (%)	5.75 ± 1.71	6.30 ± 1.03	7.70 ± 1.98	9.57 ± 1.03
Lambda_z (1/h)	0.07 ± 0.01	0.07 ± 0.004	0.07 ± 0.01	0.07 ± 0.003
Lambda_z_lower (h)	12.00	12.00	4.50 ± 1.00	5.00 ± 2.00
Lambda_z_upper (h)	36.00	36.00	36.00	36.00
AUMClast (h h ng/mL)	5334.39 ± 857.88	5832.99 ± 1047.68	1313.09 ± 231.29	1567.81 ± 393.92
AUMCINF_obs (h h ng/mL)	7173.60 ± 1563.80	7932.15 ± 1436.72	1816.69 ± 431.07	2311.14 ± 634.17
MRTlast (h)	9.33 ± 0.32	9.45 ± 0.13	11.20 ± 1.24	11.62 ± 0.28
Vz_F_obs (L)	866.37 ± 124.12	848.24 ± 176.88	4155.02 ± 441.18	4011.84 ± 890.25
AUClast/AUCINF_obs	0.94 ± 0.02	0.94 ± 0.01	0.92 ± 0.02	0.90 ± 0.01
CL_F_obs (L/h)	63.34 ± 12.98	58.38 ± 11.10	298.55 ± 34.22	262.19 ± 58.92

*T*_*max*_ Time of maximum observed concentration, *C*_*max*_ Maximum observed concentration, occurring at time T_max_, *AUClast* Area under the curve from the time of dosing to the time of the last measurable (positive) concentration, *AUCINF_obs* AUC from time of dosing extrapolated to infinity, based on the last observed concentration, *HL_Lambda_z* Terminal half-life [ln(2)/λ_z_], *AUC_%Extrap_obs* Percentage of AUCINF_obs due to extrapolation from Tlast to infinity, *Lambda_z* First-order rate constant associated with the terminal (log-linear) portion of the curve [Estimated by linear regression of time vs. log concentration], *Lambda_z_lower* Lower limit on time for values to be included in the calculation of Lambda Z, *Lambda_z_upper* Upper limit on time for values to be included in the calculation of Lambda Z, *AUMClast* Area under the moment curve from the time of dosing to the last measurable (positive) concentration, *AUMCINF_obs* Area under the first moment curve (AUMC) extrapolated to infinity, based on the last observed concentration, *MRTlast* Mean residence time from the time of dosing to the time of the last measurable concentration, *Vz_F_obs* Volume of distribution based on the terminal phase, *CL_F_obs* Total body clearance for extravascular administration, where F is the fraction of dose absorbed.

**Table 12:** Summary of statistical assurance of bioequivalent similarity of reference and test product.

Analyte	VEN			ODV		
PK parameters	C_max_ (ng/mL)	AUClast (h ng/mL)	AUCINF__obs_ (h ng/mL)	C_max_ (ng/mL)	AUClast (h ng/mL)	AUCINF__obs_ (h ng/mL)
Least square mean (T)	58.19	609.91	650.93	10.12	131.92	145.89
Least square mean (R)	57.69	566.21	600.81	9.55	116.50	126.25
Mean square error	0.05	0.03	0.04	0.02	0.03	0.03
Intersubject variability	22.50	18.79	19.18	13.77	17.43	18.57
SE of difference	0.11	0.09	0.10	0.07	0.09	0.09
Power	33.82	48.03	46.21	77.55	55.05	49.12
100 × T/R ratio	100.87	107.72	108.34	105.99	113.24	115.56
90% CI Lower limit	74.33	83.39	83.44	87.80	89.28	89.73
90% CI Upper limit	136.88	139.14	140.68	127.95	143.63	148.83

## Conclusion

The preclinical PK data obtained from small studies, with a limited number of animals and plasma samples, during the formulation development phase can offer critical PK information of a drug and its dosage form for attaining the requisite clinical PK behavior. In this work, we are reporting preclinical BE assessment studies of two ER formulations of VEN, Efexor XR, and an in-house produced VEN ER tablet, in New Zealand White rabbits. For this purpose, a reliable and rapid SPE based LC-MRM-MS/MS bioanalytical method, for the in tandem quantitation of VEN and ODV, was developed and effectively validated in agreement with the permissible limits of FDA recommendations and acceptance criteria.

## Data Availability

All data generated or analyzed during this study are included in this published article.
